# The Effect of Statin Therapy on Bone Metabolism Markers and Mineral Density: Aa GRADE-Assessed Systematic Review and Dose-Response Meta-Analysis of Randomized Controlled Trials

**DOI:** 10.34172/apb.2024.051

**Published:** 2024-06-22

**Authors:** Seyyed Mostafa Arabi, Mahla Chambari, Leila Sadat Bahrami, Ali Jafari, Hossein Bahari, Željko Reiner, Amirhossein Sahebkar

**Affiliations:** ^1^Noncommunicable Diseases Research Center, Neyshabur University of Medical Sciences, Neyshabur, Iran.; ^2^Healthy Ageing Research Centre, Neyshabur University of Medical Sciences, Neyshabur, Iran.; ^3^Department of Food Science and Nutrition, Faculty of Applied Sciences, UCSI university, 56000 Cheras, Wilayah Persekutuan Kuala Lumpur, Malaysia.; ^4^Department of Nutrition, Faculty of Medicine, Mashhad University of Medical Sciences, Mashhad, Iran.; ^5^Golestan Research Center of Gastroenterology and Hepatology, Golestan University of Medical Sciences, Gorgan, Iran.; ^6^Student Research Committee, Department of Nutrition, School of Health, Golestan University of Medical Sciences, Gorgan, Iran.; ^7^Department of Internal Medicine, University Hospital Center Zagreb, Zagreb, Croatia.; ^8^Polish Mother’s Memorial Hospital Research Institute, Lodz, Poland.; ^9^Center for Global Health Research, Saveetha Medical College and Hospitals, Saveetha Institute of Medical and Technical Sciences, Saveetha University, Chennai, India.; ^10^Applied Biomedical Research Center, Mashhad University of Medical Sciences, Mashhad, Iran.; ^11^Biotechnology Research Center, Pharmaceutical Technology Institute, Mashhad University of Medical Sciences, Mashhad, Iran.

**Keywords:** Atorvastatin, Bone markers, Bone-specific alkaline phosphatase, Osteoporosis, Meta-analysis, Statins

## Abstract

**Purpose:**

Statin therapy is widely used for the management of dyslipidemia and the prevention of cardiovascular diseases (CVDs). However, there is a growing concern about its potential effects on bone metabolism markers and mineral density. The aim of this systematic review and meta-analysis was to investigate the effect of statin therapy on these parameters.

**Methods:**

PubMed/MEDLINE, Scopus, and Clarivate Analytics Web of Science databases were searched from inception to August 2023, using MESH terms and keywords.

**Results:**

After screening 2450 articles, 16 studies that met the inclusion criteria were included, of which 12 randomized controlled trials (RCTs) were used for meta-analysis. The findings showed that statin therapy significantly reduced bone-specific alkaline phosphatase (B-ALP) levels (WMD=-1.1 U/L; 95% CI -2.2 to -0.07; *P*=0.03; I^2^=0%,), and bone mineral density (BMD) at different sites (WMD=-0.06 g/cm^2^; 95% CI -0.08 to -0.04; *P*<0.001; I^2^=97.7%, *P*<0.001). However, this treatment did not have a significant effect on osteocalcin, serum C-terminal peptide of type I collagen (S-CTx), serum N-telopeptides of type I collagen (NTx) concentration, or overall fracture risk.

**Conclusion:**

This systematic review and meta-analysis provide evidence that statin therapy is associated with a significant reduction in B-ALP levels and BMD at different sites of the skeleton. Further studies are needed to investigate the long-term effects of statin therapy on bone health and to identify the potential underlying mechanisms.

## Introduction

 Osteoporosis is a widely prevalent disease, particularly among the elderly, and it is not only a significant health problem but also a financial burden for the global healthcare system, especially in the context of the aging population in many countries.^1^ This disease is characterized by low bone mineral density (BMD) and deterioration of bone tissue, leading to an increased risk of fractures, particularly hip fractures, and mortality in adults, especially elderly.^2^ This disease is defined by BMD with a standard deviation (SD) difference equal to or less than 2.5 from ordinary high levels for fit young adults.^3^ While osteoporosis affects both men and women, it is more common in women due to their lower bone density and hormonal changes in menopause.^4^ Fractures caused by osteoporosis can result in chronic pain, disability, need for long-lasting nursing home care, and decreased quality of life. They often require hospitalization and surgery thus leading to increased healthcare costs.^5^ Prevention and management of osteoporosis require a multifaceted approach. Adequate intake of calcium and vitamin D, regular exercise, and avoidance of smoking and excessive alcohol consumption are essential for maintaining healthy bone.^4^ Therapy with bisphosphonates is considered to be the first line of pharmacological treatment for postmenopausal osteoporosis which slows down bone loss and reduces fracture risk.^6,7^ The effects of some new drugs are still evaluated e.g. monoclonal antibodies inhibiting cathepsin K, a lysosomal cysteine protease with the highest expression in osteoclasts - the cells responsible for bone resorption, and romosozumab which inhibits the activity of sclerostin, an inhibitor of bone formation, particularly in the bones of elderly people.^8^

 Some studies have shown no association between dyslipidemia, particularly hypercholesterolemia, and BMD while others have reported a negative or a positive effect for total serum cholesterol, low-density lipoprotein cholesterol (LDL-cholesterol or LDL-c), and other serum lipoproteins.^9-11^

 Statins are cholesterol-lowering drugs that, among a myriad of drug classes,^12-14^ are the most widely used drugs for the treatment of dyslipidemia and prevention of atherosclerotic cardiovascular disease (CVD).^15,16^ These drugs inhibit the activity of 3-hydroxy-3-methylglutaryl coenzyme A reductase, an enzyme that plays a key role in the synthesis of cholesterol in the liver.^17^ By reducing the activity of this enzyme, statins decrease the levels of atherogenic LDL-c in the blood, and elevated LDL-c is a major risk factor for the development of atherosclerosis and atherosclerotic CVD.^18^ Beyond cholesterol-lowering effects, statins also possess numerous pleiotropic actions.^19-26^ However, there has been a lack of consensus concerning the effect of statin therapy on bone health and osteoporosis. Some studies have suggested that statin therapy may have a positive effect on bone metabolism markers such as BMD and bone turnover markers and that they may be involved in different mechanisms including proliferation, differentiation, protection of osteoblasts, and reducing genesis of osteoclasts.^27-30^ A recently published study has revealed that treatment with statins was linked to a significant reduction in the risk of osteoporotic fractures in the general older population.^31^ Another recently published study has shown that treatment with statins in adults with type 2 diabetes mellitus was associated with a lower risk of hip fractures demonstrating a dose-response relationship.^32^

 Nevertheless, other studies have not shown such a beneficial effect.^27-29^ Even more, a recent animal study has shown that high-dose simvastatin significantly reduced bone quality in obese male and ovariectomized female mice suggesting an increased risk of osteoporosis. Such an increased risk has been also observed in a large cohort of Austrian men and women stressing that the underlying pathophysiological mechanisms are still unknown.^33^ However, in this study without considering different statin doses, men on statin therapy had a lower rate of diagnosed osteoporosis when compared to controls but in women, simvastatin therapy was associated with an increased risk of osteoporosis when compared to controls.

 The contradictory results of different studies indicate the need for further studies to determine the effect of statin therapy on bone health. The aim of this meta-analysis was to try to clarify this complex issue by systematically analyzing the accumulated evidence regarding the effects of statins on osteoporosis, specifically their effect on fracture risk, BMD, and biomarkers of bone metabolism.

## Methods and Material

 This systematic review and meta-analysis study was conducted based on Preferred Reporting Items for Systematic Reviews and Meta-Analyses (PRISMA),^34^ and the study protocol was registered at PROSPERO (registration number: CRD42023449826).

###  Intervention and control groups

 The intervention group was considered as patients who were treated with all types of statins administered orally and the control group was defined as those treated with placebo or active control (low dose statin).

###  Data sources and searches

 We searched systematically PubMed/MEDLINE, Scopus, and Clarivate Analytics Web of Science databases to collect suitable studies from inception to August 2023 using MESH terms, keywords, and text words as listed in Table S1 (Supplementary file 1). References of related reviews and articles were also screened for additional studies that might be missed by our database search.

###  Study selection

 Duplicated studies were removed, and two expert researchers (H.B. and A.J.) separately assessed studies by title and abstract to select the relative ones. Studies which met the following criteria were included: 1) parallel or crossover randomized, blinded clinical trials, 2) statin treatment of any type or dosage (except low-dose statin treatment) or duration as the intervention group, 3) placebo or low-dose statin therapy as a control group, 4) studies which reported serum osteocalcin, C terminal peptide of type I collagen (S-CTx), bone-specific alkaline phosphatase (B-ALP), serum N-telopeptides of type I collagen (NTx), BMD, and overall fracture risk, 5) studies including participants, who are using any kind of statins for any reason, 6) The participants were healthy people and patients with various health conditions such as osteopenia, osteoporosis, hypercholesterolemia, hyperlipidemia, type 2 diabetes mellitus, pre-menopausal women, patients who had total hip arthroplasty, fractures, ischemic heart disease, and individuals infected with human immunodeficiency virus (HIV).^35-50^ The participants in the included RCTs in this study were not using anabolic agents, growth hormones, osteoporosis therapy (bisphosphonates, teriparatide, calcitonin), or any medications for diseases affecting bone metabolism.

###  Data extraction and quality assessment

 Two reviewers (M.C. and A.J) checked the full text of randomized controlled trials (RCTs) and extracted the following data from the included studies: country of the study, publication year, first author’s name, study design, sample size, intervention and control group sample size, mean age, sex, statin and placebo dosage, type of statins, duration of treatment, mean changes, and standard deviations (SD) of considered outcomes for both intervention and control groups, and the confounding variables adjusted in the analyses. The risk of bias assessment was done by Cochrane’s risk of bias assessment tool 1 (ROB1) which consists of the following domains: random sequence generation, allocation concealment, reporting bias, performance bias, detection bias, attrition bias, and other sources of bias.^51^ Based on a total score of domains the RCTs were considered as good (when less than two domains had a high risk of bias), fair (when two domains had a high risk of bias), or poor (when two or more than two domains had high risk of bias).^51^

###  Statistical assessment

 The meta-analysis was conducted using weighted mean difference (WMD) and SD of outcomes to standardize between-group differences, and the random effect model was used because of study heterogeneity.^52^ The heterogeneity of studies was calculated with the I^2^ test, and I^2^ more than 50% was considered as high heterogeneity.^52^ To find the source of heterogeneity subgroup analysis of age, sex, statin type, duration of treatment, study quality, and statin solubility (lipophilic or hydrophilic) was conducted, and a meta-regression test of statin therapy based on the duration of intervention was performed. A nonlinear dose-response of atorvastatin, Egger regression test for publication bias assessment, influence analysis, and GRADE assessment (Grading of Recommendations, Assessment, Development, and Evaluations) were also done. Results were considered significant when *P* < 0.05 and the meta-analysis was performed using STATA software (StataCorp, College Station, Texas, USA).

## Results and Discussion

###  Result of the search

**Figure 1:**
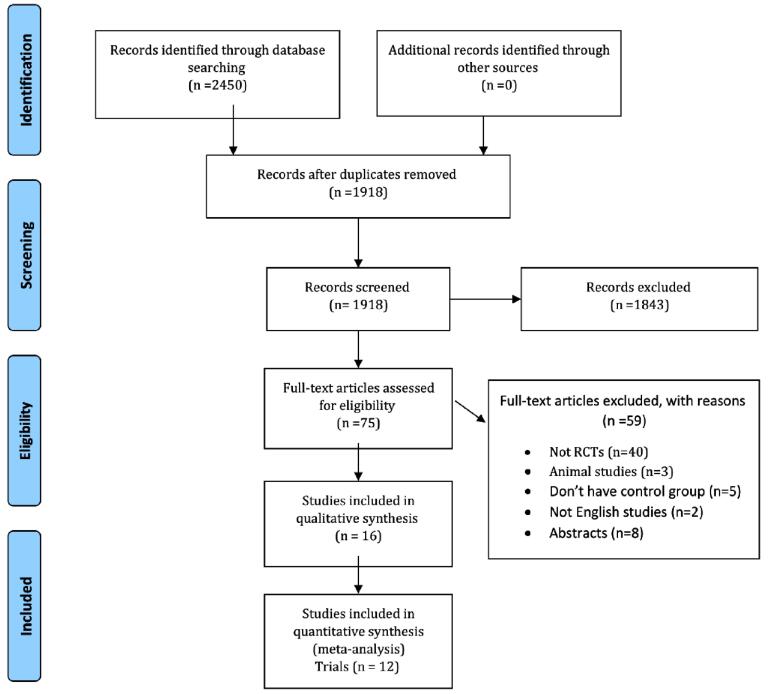
PRISMA flow diagram

 A total of 2450 articles were found in the initial literature search, but only 16 studies met the inclusion criteria,^35-50^ of these, 12 RCTs were used for meta-analysis as shown in Figure 1.^35-42,45,46,48,49^ Table 1 shows the characteristics of the included trials and patients. The included studies in this systematic review were conducted in the USA, Denmark, China, Germany, Turkey, Thailand, Australia, and the UK between 2000 and 2019.^35-50^ The number of patients ranged from 16 to 17802 participants with a mean age ranging from 46.2 to 80.08 years. The statins used in the studies included atorvastatin, simvastatin, rosuvastatin, pravastatin, and fluvastatin with durations of treatment ranging from 8 to 96 weeks. Eight of the included RCTs used a double-blind study design, while 2 of them used a single-blind design.^35-50^ The quality of studies was evaluated using ROB1.^51^ According to these 2 studies were of good quality,^37,39^ 9 studies were of fair quality,^38,40-42,45,46,48-50^ and 5 studies were of poor-quality category^35,36,43,44,47^ (Table S2, Supplementary file 1). To maintain the integrity of our meta-analysis, we decided to exclude the studies by Zhang et al and Erlandson et al.^43,47,50^ In the case of Zhang and colleagues’ study, the data was presented in different units, which required the use of the SMD method to pool the results. Unfortunately, this led to the results that could not be accepted. Despite trying to contact the authors for clarification, we did not receive any response. As a result, we decided that it was necessary to remove this study from the meta-analysis. In the case of Erlandson’s study, the mean and standard deviation change could not be calculated, making it inappropriate for meta-analysis. Therefore, this study was also excluded from this meta-analysis.

**Table 1:** Characteristics of included studies

**First author, year, country**	**Design**	**Design**	**Participants (n)** **Int/con**	**Age means (year)** **Int/con**	**Intervention**			**Duration of intervention (wk)**	**Outcomes (Change)**	
					**Treatment group**	**Control group**	**dose** **(mg/d)**		**Treatment group**	**Control group**
Bjarnason,^35^ 2001, Denmark	RCT	43/21	Normal premenopausal	71.2/71.1	Fluvastatin + Vitamin C	Vitamin C	40	14	CTX: -148 ± 384	Bjarnason,2001, Denmark
Hsia,^36^ 2002, USA	DB, RCT	8/8	Osteopenia	56.1/56.1	Simvastatin	Placebo	20	12	CTX:16.9 ± 34.82	-8.5 ± 21.6
							40		12.5 ± 21.5	-8.5 ± 21.6
									ALP: -1.8 ± 5.21	0.1 ± 4.9
									-0.3 ± 4.14	0.1 ± 4.9
									NTX:3.1 ± 3.13	0.4 ± 3.05
									-1.2 ± 4.07	0.4 ± 3.05
Rejnmark,^37^ 2004, Denmark	DB, RCT	39/39	Normal premenopausal	63/64	Simvastatin	Placebo	40	52	BMD LS:0.006 ± 0.05	0.006 ± 0.06
									TH:0 ± 0.05	0.001 ± 0.05
									FN:0.002 ± 0.04	0.003 ± 0.05
									IT:0.023 ± 0.07	0.002 ± 0.06
									T:0.007 ± 0.05	0.007 ± 0.05
									W: -0.025 ± 0.05	-0.001 ± 0.05
Bone,^38^ 2007, USA	DB, RCT			59/67	Atorvastatin	Placebo	10	52	BMD: -0.23 ± 0.23	0.1 ± 0.2
				65/67			20		-0.34 ± 0.21	0.1 ± 0.2
				67/67			40		-0.4 ± 0.23	0.1 ± 0.2
				60/67			80		-0.02 ± 0.24	0.1 ± 0.2
Berthold,^39^ 2004, Germany	DB, RCT	24/25	Normal premenopausal	62.7/60.1	Atorvastatin	Placebo	20	8	CTX:0.021 ± 0.19	0.02 ± 0.1
									ALP: -0.8 ± 4.19	1.5 ± 3.7
Braatvedt,^40^ 2004, New Zealand	Crossover, RCT	25/25	Type 2 diabetes mellitus	56/56	Atorvastatin	Placebo	40	12	CTX: -0.02 ± 0.09	-0.02 ± 0.1
									Osteocalcin: -1.27 ± 5.7	0.3 ± 5.1
									ALP: -1.46 ± 5.79	-0.31 ± 5.9
Rosenson,^41^ 2005, USA	DB, RCT	12/14	Healthy nonsmoking adults	51.3/50.1	Pravastatin	Placebo	40	8	Osteocalcin: 0.1 ± 1.68, 0.6 ± 1.08, -0.8 ± 1.81	-0.1 ± 0.9, -0.1 ± 0.9, -0.1 ± 0.9, 0.7 ± 3.7, 0.7 ± 3.7, 0.7 ± 3.7, 0.2 ± 2.04, 0.2 ± 2.04, 0.2 ± 2.04
		14/14		51/50.1					ALP:0.4 ± 3.05, -0.1 ± 4.47, -3 ± 5.7	
		15/14		49.6/50.1					NTX: -1.2 ± 7.13, 0.1 ± 2.41, -0.9 ± 2.05	
Tanriverdi,^42^ 2005, Turkey	SB, RCT	57/58	Hypercholesterolemia postmenopausal	54/54.7	Atorvastatin + Risedronate	Risedronate	20	24	ALP: -4.07 ± 11.22 risedronate plus atorvastatin produced significantly greater increases in the bone mineral density of the lumbar spine (1.58% versus 0.75%, *P* < 0.05).	-1.37 ± 11.9
Zhang,^43^ 2018, China	RCT, SB	21/21	Hypercholesterolemia with total hip arthroplasty	69.4/68.6	Simvastatin	Placebo	40	48	The loss of BMD in ROIs 3 and 5 was only, significantly observed at three months follow-up and recovered thereafter. There were no significant detected changes of BMD in ROI 4.	In the control group, patients showed significant loss of periprosthetic BMD in ROIs 1, 2, 6, and 7 throughout the study period.
Chen,^44^ 2014, China	RCT	32/32	Elderly males with osteopenia	80.8/79.3	Atorvastatin	Placebo	10	48	BMD TH:0.01 ± 0.01	-0.01 ± 0.01
									FN:0 ± 0.01	0 ± 0.01
									LS:0 ± 0.01	0 ± 0.01
Chuengsamarn,^45^ 2010, Thailand	RCT	81/80	Hyperlipidemia	62.15/61.65	Simvastatin	Gemfibrozil	40	72	BMD:0.045 ± 0.057 0.29 ± 0.2	-0.01 ± 0.04 0.23 ± 0.2
Patil,^46^ 2009, UK	DB, RCT	31/31	Fracture	56.5/57.3	Simvastatin	Placebo	20	12	CTX:0 ± 0.08	0.04 ± 0.08
									Osteocalcin:1.8 ± 5.84	1.3 ± 6.02
									ALP:2.3 ± 6.41	1.5 ± 4.04
									NTX:6.7 ± 44.19	20 ± 31.6
Zhang,^47^ 2019, China	RCT, DB	54/54	Elderly with osteoporosis	65.81/65.74	Atorvastatin	Placebo	70	24	Simvastatin caused no changes in BMD	
Reid,^48^ 2000, Australia	RCT, DB	4512/4502	Ischemic heart disease	62/62	Pravastatin	Placebo	40	6 years	Fracture: HR: 0.94, UI: 0.77. LI: 1.16	
Peña,^49^ 2015, USA	RCT, DB	8901/8901	Elders with hs-CRP level of at least 2 mg/L	66/66	Rosuvastatin	Placebo	20	5 years	Fracture: HR:0.73, UI:0.88, LI:1.28	
Erlandson,^50^ 2016, USA	RCT, DB	72/75	HIV-infected individuals	45.6/46.9	Rosuvastatin	Placebo	10	96	There were no significant differences in the relative changes of BMD (*P* > 0.29)	

Legends: RCT: randomized clinical trial, F: female, M: male, Int: intervention, Con: control, LS: lumbar spine, TH: total hip, FN: femoral neck, IT: Intertrochanter, T: Trochanter, ALP: Alkaline phosphatase, CTX: C terminal peptide of type I collagen, NTX: N-telopeptides of type I collagen, BMD: bone mineral density.

###  Efficacy outcomes

####  Bone metabolism markers: Serum osteocalcin

**Figure 2:**
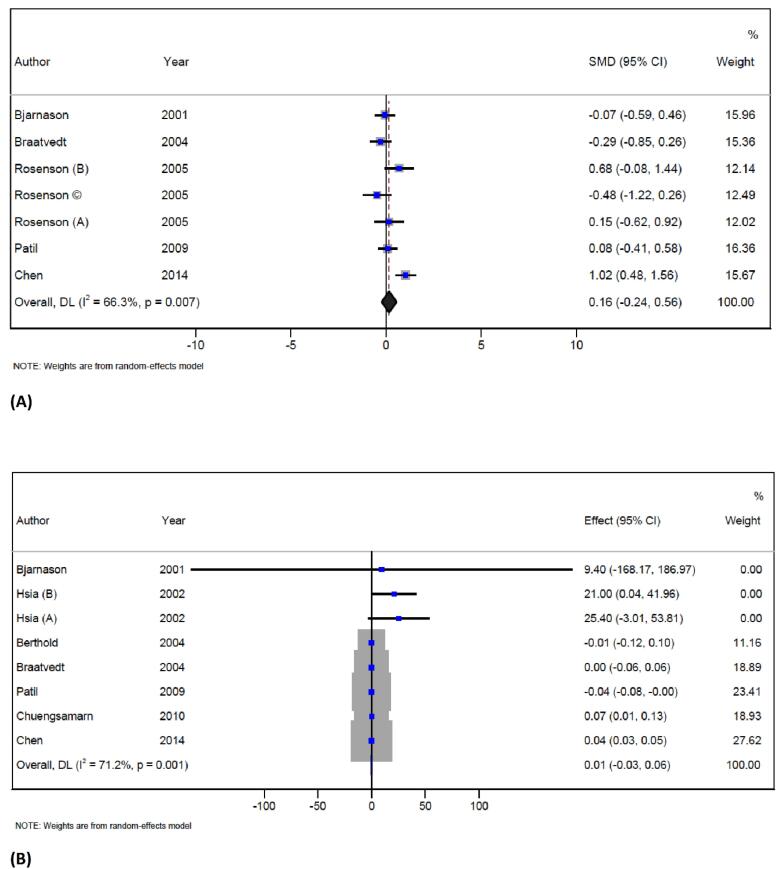
Forest plot displaying weighted mean difference and 95% confidence intervals for the effect of statin treatment on osteocalcin (A), and serum CTx (B) in trials

 In total, 5 trials with 7 arms (with 170 participants in the intervention group and 149 patients in the control group) reported data on serum osteocalcin concentration.^35,40,41,44,46^ Statin therapy did not reduce the osteocalcin level when compared to the controls (SMD = 0.1; 95% CI -0.2 to 0.5; *P* = 0.4; I^2^ = 66.3%, *P* = 0.007) (Figure 2A). However, significant heterogeneity was observed in the overall endpoint, and the source of it could be explained by study design, follow-up duration, and mean age of participants. In trials conducted in China on men, statin therapy increased serum osteocalcin. The results of the influence analysis indicate that this was not a significant effect. The results of the subgroup analysis are presented in Table S3 (Supplementary file 1).

 The literature review of earlier studies has indicated that the treatment with statins did not have a harmful effect on bone health in most observational studies.^28,53,54^ Some studies have even reported a positive effect of statin therapy on osteoblastic markers such as osteocalcin.^27,37^ There are specific pathways that have been identified as having a potential role concerning the effect of statin treatment on bone anabolism. It has been shown that statins could increase levels of bone morphogenetic protein-2 (BMP-2) through the Ras-PI3K-Akt/MAPK signaling pathway, which then triggers osteoblast differentiation via Runt-related transcription factor 2 (Runx2).^55^ Moreover, statins interfere with the mevalonate pathway, thereby inhibiting the synthesis of downstream products such as steroids, vitamin D, and coenzyme Q10, which negatively control osteoblastic differentiation.^56-59^ It seems that statins might inhibit osteoblast apoptosis through the TGFβ/Smad3 pathway and suppress osteoclast genesis via OPG/RANKL/RANK pathway.^60^

 Despite these promising findings, more recent studies have reported disappointing effects of statins on bone health.^61,62^ A study by Burden et alshowed that the treatment with statins was associated with a 3.62-fold increased risk of being diagnosed with osteoporosis.^29^ Other studies have found that lower doses of some statins were associated with a decreased risk of osteoporosis, while higher doses of other statins were associated with an increased risk.^28,49,63^

####  Bone metabolism markers: serum C terminal peptide of type I collagen (S-CTx)

 The study included 7 studies with 8 arms, involving 293 participants in the intervention group and 249 subjects in the control group.^35,36,39,40,44-46^ These trials reported data on S-CTX concentration, and the results showed that statin therapy did not change the S-CTX concentration when compared to the controls (WMD = 0.01 ng/mL; 95% CI -0.03 to 0.06; *P* = 0.5; I^2^ = 71.2%, *P* = 0.001) (Figure 2B). However, there was significant heterogeneity in the overall endpoint, which could be attributed to statin types, location of the trials, follow-up and duration of treatment as well as the mean age of participants.

 Subgroup analysis based on statin type has shown that atorvastatin therapy increased the S-CTX concentration. The analysis based on three locations – studies performed in the USA, Thailand, and China has shown that statin therapy increased S-CTX levels. Moreover, the analysis showed that statin therapy significantly increased CTX concentration in males, when the duration of intervention was more than 12 weeks as well as in participants older than 60 years. The results of the subgroup analysis are presented in Table S3 (Supplementary file 1). The influence analysis showed that no individual study had a significant effect on the pooled effect size, and no publication bias was detected by Egger’s test and funnel plot (Supplementary file 1, Figure S1).

 This meta-analysis could not confirm any effect of statins on S-CTx. S-CTx is a substance that is released during bone resorption and it has been shown in some studies that higher values of S-CTx were associated with the lower spine, hip, and femur BMD at baseline.^64^ The reason of the no effect in the present meta-analysis might be that S-CTx has a significant biological variation due to circadian rhythm and diet. However, although S-CTx is used to predict fracture risk independently of BMD, the lack of data is the cause that it is not included in fracture risk calculators.^65^ It is interesting to mention that an earlier study has shown a positive correlation between S-CTx and blood cholesterol.^66^

####  Bone metabolism markers: bone-specific alkaline phosphatase (B-ALP)

**Figure 3:**
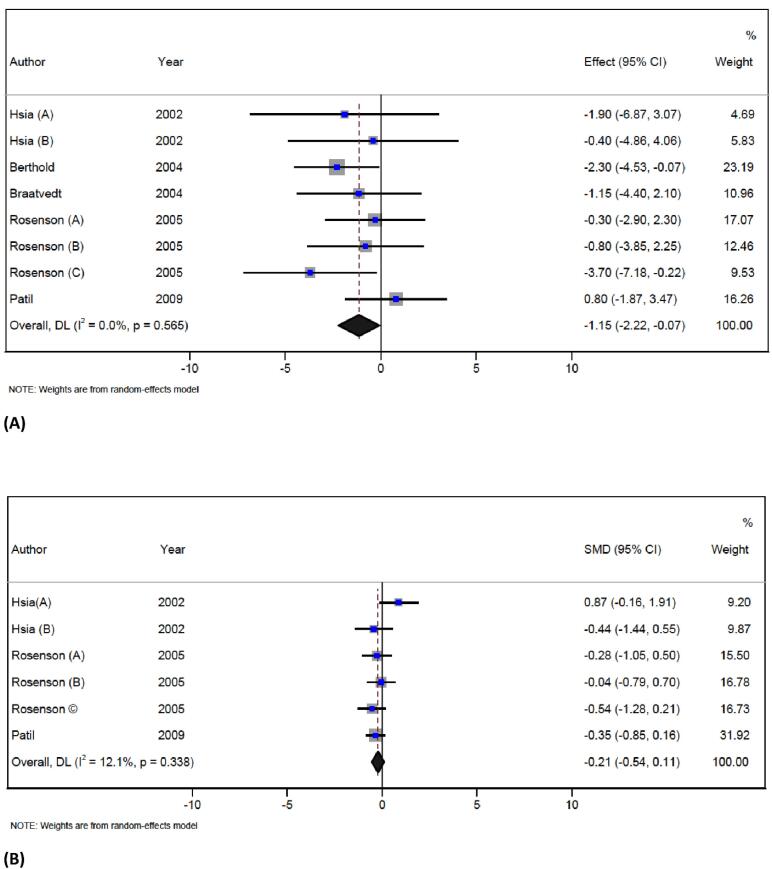
Forest plot displaying weighted mean difference and 95% confidence intervals for the effect of statin treatment on serum ALP (A), and serum NTx (B) in trials

 The pooled analysis of 5 papers with 8 arms and a total of 391 participants indicates that statin therapy significantly reduces BALP concentration (WMD = -1.1 U/L; 95% CI -2.2 to -0.07; *P* = 0.03) without any significant heterogeneity observed across the studies (I^2^ = 0%, *P* = 0.5) (Figure 3A). The influence analysis showed that no individual study had a significant effect on the pooled effect size.^36,39-41,46^ The results of a subgroup analysis indicate that female participants, adults over the age of 60 who were treated with atorvastatin and were part of studies with a double-blind design and good quality, had significantly reduced B-ALP levels. This is important in the context of a most recent study which showed that the increased level of B-ALP was associated with decreased lumbar BMD in middle-aged adults.^67^ The findings of the present meta-analysis suggest that statin therapy may be effective in reducing bone remodeling rates. The underlying mechanisms for this decrease in B-ALP levels are not yet fully understood, but there is a possibility that it might be related to the mevalonate pathway.^29^ An earlier meta-analysis has shown that statin therapy did not affect B-ALP levels but that it could increase the concentration of osteocalcin.^27^ However, the present meta-analysis could not confirm any significant effect of statins on osteocalcin. The differences between the results of this meta-analysis and those of an earlier meta-analysis by An et al may be due to the differences in the methodology used such as the pooled method or the selection of included studies.^27^ In the meta-analysis by An et al the selection criteria differed from those used in this meta-analysis since they included two articles - one that was focused on combination therapy and the other that had an active control (ezetimibe).^27^ This meta-analysis, on the other hand, had a different approach to study selection.

####  Bone metabolism markers: serum N-telopeptides of type I collagen (NTx)

 In total, 3 trials with 6 arms (with 88 participants in the intervention group and 89 patients in the control group) reported data on serum NTx concentration.^36,41,46^ Statin therapy did not reduce the NTx level when compared to the control group (SMD = -0.2; 95% CI -0.5 to 0.1; *P* = 0.1; I^2^ = 12.1%, *P* = 0.3) (Figure 3B). The influence analysis showed that the overall effect size was not significantly changed by any individual study.

 This meta-analysis could not prove any effects of statins on serum NTx which are considered to be indicators of bone resorption since they are important in the collagen degradation process.^68^ NTx was in some studies associated with densitometry T-score of the spine and hip at baseline. Since it is a marker of resorption, its levels may be increased in increased bone turnover, leading to a reduction in BMD but as mentioned already, this meta-analysis could not prove any significant effect of statins on it.

###  The BMD at different sites of the skeleton and fractures 

**Figure 4:**
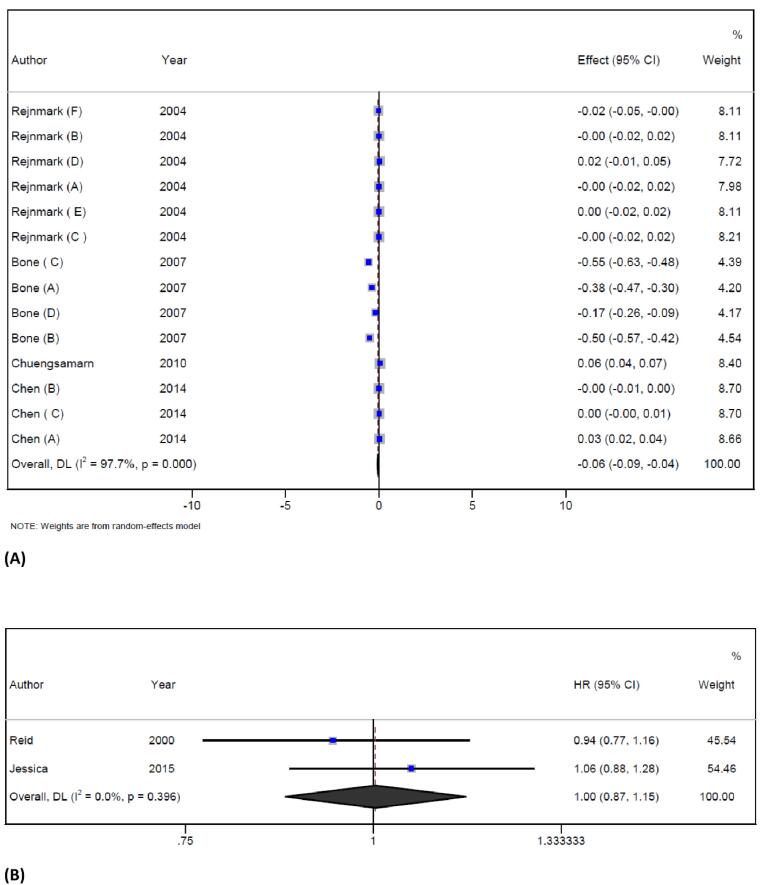
Forest plot displaying weighted mean difference and 95% confidence intervals for the effect of statin treatment on BMD (A), and fracture (B) in trials

 The pooled analysis of 4 trials^37,38,44,45^ with 14 arms reported data for BMD. Statin therapy decreased overall BMD levels (WMD = -0.06 g/cm^2^; 95% CI -0.08 to -0.04; *P* < 0.001; I^2^ = 97.7%, *P* < 0.001) (Figure 4A). Six studies reported data for BMD of lumbar spine and showed that statin treatment reduced BMD (WMD = -0.2 g/cm^2^; 95% CI -0.3 to -0.1; *P* < 0.001; I^2^ = 98.9%). However, one study reported data for BMD of the forearm and showed that statin therapy improved the BMD (WMD = 0.01 g/cm^2^; 95% CI -0.03 to 0.04; *P* = 0.2; I^2^ = 84.2%). Finally, statin therapy did not have any effect on BMD of the femoral neck (WMD = -0.002 g/cm^2^; 95% CI -0.007 to 0.003; *P* = 0.4; I^2^ = 0%), the intertrochanteric region (WMD = 0.02 g/cm^2^; 95% CI -0.008 to 0.05; *P* = 0.1; ), the total hip (WMD = 0.01 g/cm^2^; 95% CI -0.03 to 0.04; *P* = 0.2; I^2^ = 84.2%), the trochanter (WMD = 0.001 g/cm^2^; 95% CI -0.02 to 0.02; *P* = 0.99), and the forearm (WMD = 0.05 g/cm^2^; 95% CI 0.04 to 0.07; *P* < 0.001). Significant heterogeneity was observed in the overall endpoint, and the source of it could be explained by statin types, the location of the site of the skeleton, and the overall quality of included studies. Subgroup analysis based on three different sites of the skeleton conducted in the USA has shown that statin therapy reduced BMD and the effect of statin therapy on BMD was more pronounced in females than in males. This study also found that participants who were under 60 years old had a more significant reduction in BMD when compared to those who were older. The quality of the included trials was also found to be a significant factor in determining the effect of statin therapy. The study found that trials with fair quality showed a more significant effect on BMD than those with good quality. Finally, atorvastatin therapy had a significant effect on BMD when compared with other statins. The results of the subgroup analysis are presented in Table S3. The pooled analysis of 2 trials^48,49^ showed that the overall risk of fracture following statin therapy was not significant when compared with the control group (HR = -1.004; 95% CI 0.8 to 1.1; *P* = 0.9; I2 = 0%, *P* = 0.3) (Figure 4B). The influence analysis showed that no individual study had a significant effect on the pooled effect size, and no publication bias was detected by Egger’s test and funnel plot (Figure S1).

 The effect of statin treatment on bone reabsorption markers remains uncertain. Previous studies have reported different results of treatment with statins on BMD at different sites of the skeleton. An earlier meta-analysis has shown that statin treatment causes a higher BMD at the lumbar spine and total hip when compared to baseline levels, but no such effect has been showed at the femoral neck.^27^ One possible explanation for these different results might be that statins may affect bone metabolism differently depending on the site of the skeleton.^37^ This is different from the data on bisphosphonates which clearly improve BMD and decrease bone turnover markers even in women in early menopause.^69^ Although the previously mentioned meta-analysis did not show a significant effect on overall BMD scores,^27^ the present meta-analysis has indicated that statin therapy can reduce overall BMD. Furthermore, the subgroup analysis has shown that BMD was significantly decreased in the lumbar spine, in women, in adults under 60 years, and in those who were treated with statins for more than 50 weeks. These variations in BMD across distinct skeletal sites may be associated with different reactions to various pathological conditions. For instance, cortical BMD was reduced more than trabecular BMD in hyper-parathyroid patients.^70^ The lack of positive effects on lumbar spine BMD in female patients may be due to decreased osteoblastic function in males and increased bone resorption related to reduced estrogen levels in females.^71^ However, it is possible that statins cannot entirely compensate for bone loss related to estrogen loss. The low uptake of statins into the bone and their low bioavailability in bone may be responsible for the lack of significant effects at some endpoints.^72^ The results of the present meta-analysis indicate that there is a significant heterogeneity in the overall BMD level. Although this finding might be important, it should be interpreted with caution. The results of the dose-response analysis indicated that treatment with atorvastatin was associated with a reduction in BMD. Specifically, at a dose of 20 mg, a significant reduction in BMD was observed, which has been confirmed by several studies.

###  Meta-regression

 A meta-regression analysis was conducted to investigate the relationship between the duration of statin therapy and BMD. The results of this analysis showed that the duration of statin therapy did not have any significant effect on BMD (WMD = 0.004 g/cm^2^; 95% CI -0.01 to 0.02; *P =*0.6) (Supplementary file 1, Figure S2).

###  Non-linear dose-response meta-analysis

 Although the BMD score showed a decreasing trend with increasing doses, the findings of this study indicate that there was no significant relationship between dose and response. At a dosage of 20 mg of atorvastatin, a significant reduction in BMD was observed (MD: -0.2 g/cm^2^, 95% CI: -0.4 to -0.01), which is presented in Figure S3 (Supplementary file 1).

###  Certainty of evidences 

 Based on the GRADE evaluations, the level of evidence for ALP, BMD, NTX, and osteocalcin was rated as moderate. However, the level of evidence for CTX was rated as low. This indicates that the available studies on ALP, BMD, NTX, and osteocalcin provide a moderate level of confidence concerning their effectiveness, while the studies on CTX provide a relatively lower level of confidence. Details of the GRADE analysis are presented in Table S4(Supplementary file 1).

## Strengths and limitations

 To the best of our knowledge, the present systematic review and meta-analysis is the first comprehensive analysis of the effect of statin therapy on bone metabolism markers and BMD levels in RCT studies. Nevertheless, this meta-analysis has some limitations First, the majority of the studies included in this analysis were not of high quality. Second, due to the limited number of studies available for analysis, advanced statistical assessments could not be performed. However, the present analysis showed that the certainty of evidence for the majority of results was moderate.

## Conclusion

 The systematic review and meta-analysis of different trials indicated that statin therapy could potentially reduce the levels of B-ALP and BMD. These findings suggest that statin therapy may have a beneficial on bone metabolism but also an opposite effect as well. Therefore, further studies are required to prove the long-term effects of statin therapy on bone health. It is important to stress that while the use of statins may have complex effects on the bone, the benefits of these drugs in reducing the risk of atherosclerotic CVD by far surpass any possible adverse effects on the bone if they exist at all.

 The results of this meta-analysis might have implications for clinicians who are treating patients with osteoporosis or other bone-related conditions.

## Acknowledgments

 SM. Arabi, Z. Reiner, and A. Sahebkar were involved in the study’s conceptualization, literature search, data extraction, data analysis, and manuscript preparation. H. Bahari, A. Jafari, and M. Chambari contributed to the literature search, data extraction, and manuscript drafting. SM. Arabi, LS. Bahrami and M. Chambari contributed to the study conception and manuscript drafting. LS. Bahrami, Z. Reiner, and A. Sahebkar critically revised the manuscript. All authors take full responsibility for the analyses and interpretation of the report. All authors read and approved the final manuscript. This research did not receive any specific grant from funding agencies in the public, commercial, or not-for-profit sectors.

## Competing interests

 All authors declare that they have no conflicts of interest.

## Declaration of AI and AI-assisted technologies

 During the preparation of this work, the author(s) used the Monica AI and Grammarly tools to enhance the English language proficiency of this manuscript. By using this tool, we can effectively shorten the sentences and ensure that the writing is clear and concise. This enhances the readability of the manuscript and makes it more accessible to a wider audience. After using this tool, the author(s) reviewed and edited the content as needed and take(s) full responsibility for the content of the publication.

## Ethical Approval

 Not applicable.

## Supplementary Files

Supplementary file 1 contains Table S1-S4 and Figure S1-S3.
