# Supplementary material for: The Effect of Statin Therapy on Bone Metabolism Markers and Mineral Density: Aa GRADE-Assessed Systematic Review and Dose-Response Meta-Analysis of Randomized Controlled Trials
Source: Adv Pharm Bull. 2024 Jun 22;14(3):591–603. doi: 10.34172/apb.2024.051 (PMC11530883; doi:10.34172/apb.2024.051)
Supplement: Supplementary file 1 — contains Table S1-S4 and Figure S1-S3. [file apb-14-591-s001.pdf]

Adv Pharm Bull, 2024, 14(3), 591-S1
doi: 10.34172/apb.2024.051
https://apb.tbzmed.ac.ir

Table S1. Search strategy to find potential eligible trials for inclusion in a meta-analysis of Search strategy:

PubMed: 895

Scopus: 983

Web of Science: 572

Total: 2450

**Database**	
**PubMed**<br><br>Results:895	bone[Title/Abstract]) OR ("hip fracture"[Title/Abstract])) OR ((Osteoporosis[MeSH Terms]) OR ((((((Osteoporosis[Title/Abstract]) OR (Osteomalacia[Title/Abstract])) OR ("Low bone density"[Title/Abstract])) OR ("bone mineral density"[Title/Abstract])) OR (BMD[Title/Abstract])) OR (Osteodystrophy[Title/Abstract])))) AND (Statin[Title/Abstract] OR atorvastatin[Title/Abstract] OR Lipitor[Title/Abstract] OR fluvastatin[Title/Abstract] OR lovastatin[Title/Abstract] OR pravastatin[Title/Abstract] OR rosuvastatin[Title/Abstract] OR simvastatin[Title/Abstract] OR pitavastatin[Title/Abstract] OR Livalo[Title/Abstract OR Zocor[Title/Abstract] OR Crestor[Title/Abstract] OR Pravachol[Title/Abstract] OR Altoprev[Title/Abstract] OR Lescol[Title/Abstract] OR statins[MeSH] OR statin[MeSH])) AND ("interventive"[All Fields] OR "methods"[MeSH Terms] OR "methods"[All Fields] OR "intervention"[All Fields] OR "interventional"[All Fields] OR "Intervention Study"[All Fields] OR "Intervention Studies"[All Fields] OR "intervention*"[All Fields] OR "controlled trial"[All Fields] OR ("random allocation"[MeSH Terms] OR ("random"[All Fields] AND "allocation"[All Fields]) OR "random allocation"[All Fields] OR "randomization"[All Fields] OR "randomized"[All Fields] OR "random"[All Fields] OR "randomisation"[All Fields] OR "randomisations"[All Fields] OR "randomise"[All Fields] OR "randomised"[All Fields] OR "randomising"[All Fields] OR "randomizations"[All Fields] OR "randomize"[All Fields] OR "randomizes"[All Fields] OR "randomizing"[All Fields] OR "randomness"[All Fields] OR "randoms"[All Fields]) OR ("random allocation"[MeSH Terms] OR ("random"[All Fields]

	AND "allocation"[All Fields]) OR "random allocation"[All Fields] OR "randomization"[All Fields] OR "randomized"[All Fields] OR "random"[All Fields] OR "randomisation"[All Fields] OR "randomisations"[All Fields] OR "randomise"[All Fields] OR "randomised"[All Fields] OR "randomising"[All Fields] OR "randomizations"[All Fields] OR "randomize"[All Fields] OR "randomizes"[All Fields] OR "randomizing"[All Fields] OR "randomness"[All Fields] OR "randoms"[All Fields]) OR ("random allocation"[MeSH Terms] OR ("random"[All Fields] AND "allocation"[All Fields]) OR "random allocation"[All Fields] OR "randomization"[All Fields] OR "randomized"[All Fields] OR "random"[All Fields] OR "randomisation"[All Fields] OR "randomisations"[All Fields] OR "randomise"[All Fields] OR "randomised"[All Fields] OR "randomising"[All Fields] OR "randomizations"[All Fields] OR "randomize"[All Fields] OR "randomizes"[All Fields] OR "randomizing"[All Fields] OR "randomness"[All Fields] OR "randoms"[All Fields]) OR "randomly"[All Fields] OR ("placeboes"[All Fields] OR "placebos"[MeSH Terms] OR "placebos"[All Fields] OR "placebo"[All Fields]) OR "clinical trial"[All Fields] OR ("clinical trials as topic"[MeSH Terms] OR ("clinical"[All Fields] AND "trials"[All Fields] AND "topic"[All Fields]) OR "clinical trials as topic"[All Fields] OR "trial"[All Fields] OR "trial s"[All Fields] OR "trialed"[All Fields] OR "trialing"[All Fields] OR "trials"[All Fields]) OR "randomized controlled trial"[All Fields] OR "randomized clinical trial"[All Fields] OR "RCT"[All Fields] OR ("blinded"[All Fields] OR "blinding"[All Fields] OR "blinds"[All Fields] OR "visually impaired persons"[MeSH Terms] OR ("visually"[All Fields] AND "impaired"[All Fields] AND "persons"[All Fields]) OR "visually impaired persons"[All Fields] OR "blind"[All Fields] OR "blindness"[MeSH Terms] OR "blindness"[All Fields]) OR "double blind"[All Fields] OR "double blinded"[All Fields] OR "clinical trials"[All Fields] OR ("clinical trials as topic"[MeSH Terms] OR ("clinical"[All Fields] AND "trials"[All Fields] AND "topic"[All Fields]) OR "clinical trials as topic"[All Fields] OR "trial"[All Fields] OR "trial s"[All Fields] OR "trialed"[All Fields] OR "trialing"[All Fields] OR "trials"[All Fields]) OR "Pragmatic Clinical Trial"[All Fields] OR "Cross-Over Studies"[All Fields] OR "Cross-Over"[All Fields] OR "Cross-Over Study"[All Fields] OR ("parallel"[All Fields] OR "paralleled"[All Fields] OR "paralleling"[All Fields] OR "parallelism"[All Fields] OR "parallelisms"[All Fields] OR "parallelization"[All Fields] OR "parallelizations"[All Fields] OR "parallelize"[All Fields] OR "parallelized"[All Fields] OR "parallelizes"[All Fields] OR "parallelizing"[All Fields] OR "parallelled"[All Fields] OR "parallels"[All Fields]) OR "parallel study"[All Fields] OR "parallel trial"[All Fields])

Table S2. Quality of trials included in the meta-analysis of Statin intervention on metabolism markers and mineral density.

Study	Random sequence generation	Allocation concealment	Selective reporting	Other sources of bias	Blinding (participants and personnel)	Blinding (outcome assessment)	Incomplete outcome data	Overall Quality
**Bjarnason et al** [35]	L	L	H	H	H	H	L	Poor
**Hsia et al** [36]	L	L	H	H	L	L	U	Poor
**Rejnmark et al** [37]	L	L	L	L	L	L	L	Good
**Bone et al** [38]	L	L	H	L	L	L	H	Fair
**Berthold et al** [39]	L	L	L	L	L	L	L	Good
**Braatvedt et al** [40]	L	H	L	H	L	L	L	Fair
**Rosenson et al** [41]	L	H	L	L	L	L	L	Fair
**Tanriverdi et al** [42]	L	L	L	H	H	L	L	Fair
**Zhang et al** [43]	L	H	L	H	H	L	L	Poor
**Chen et al** [44]	L	L	L	H	H	H	L	Poor
**Chuengsamarn et al** [45]	L	H	L	H	L	L	H	Fair
**Patil et al** [46]	L	L	L	L	L	L	L	Fair
**Zhang et al** [47]	L	H	L	H	H	H	L	Poor
**Reid et al** [48]	L	H	L	L	L	L	L	Fair
**Jessica et al** [49]	L	H	L	L	L	L	L	Fair
**Erlandson et al** [50]	L	L	L	H	L	U	L	Fair

Abbreviations: **L:** low risk, **H:** high risk, **U:** unclear

Table S3. Description of subgroup analyses of Statin intervention on metabolism markers and mineral density.

	NO	WMD (95%CI)	*P*-value	heterogeneity		
				P heterogeneity	I^2	P between sub-groups
**Subgroup analyses of statin intervention on ALP**						
**Overall effect**	8	-1.1 (-2.2, -0.07)	**0.03**	0.5	0%	
**Trial location**						
USA	5	-1.2 (-2.7, 0.2)	**0.1**	0.6	0%	0.3
Germany	1	-2.3 (-4.5, -0.06)	**0.04**	-	-	
New Zealand	1	-1.1 (-4.3, 2.09)	**0.4**	-	-	
UK	1	0.8 (-1.8, 3.4)	**0.5**	-	-	
**Study design**						
Parallel	7	-1.1 (-2.2, -0.05)	**0.04**	0.4	0%	0.9
Cross-over	1	-1.1 (-4.3, 2.09)	**0.4**	-	-	
**Trial duration (weeks)**						
≤12	8	-1.1 (-2.2, -0.07)	**0.03**	0.5	0%	-
>12	0	-	**-**	-	-	
**Sex**						
Both sexes	5	-0.7 (-2.1, 0.5)	**0.2**	0.3	5.3%	0.9
Female	3	-1.9 (-3.7, -0.06)	**0.04**	0.7	0.0%	
Male	0	-	**-**	-	-	
**Baseline age (yrs)**						
<60	7	-0.7 (-2.02, 0.4)	**0.20**	0.6	0%	0.2
>60	1	-2.3 (-4.5, -0.06)	**0.04**	-	-	
**Baseline BMI (kg/m^2)**						
Normal (18.5-24.9)	11	6.6 (-4.7, 18.1)	**0.25**	0.01	56.7%	0.81
Overweight (25-29.9)	12	1.8 (-7.4, 11.0)	**0.69**	0.09	36.7%	
**Quality of studies**						
Poor	2	-1.06 (-4.3, 2.2)	**0.5**	0.6	0.0%	0.5
Fair	5	-0.7 (-2.1, 0.5)	**0.2**	0.3	5.3%	
Good	1	-2.3 (-4.5, -0.06)	**0.04**	0.26	-	
**Solubility**						
Lipophilic	7	-1.3 (-2.5, -0.1)	**0.02**	0.5	0%	0.4
Hydrophilic	1	-0.3 (-2.9, 2.3)	**0.8**	-	-	
**Statin type**						
Simvastatin	5	-0.9 (-2.5, 0.6)	**0.99**	0.3	5.9%	0.5
Atorvastatin	2	-1.9 (-3.7, -0.09)	**0.69**	0.5	0%	
Pravastatin	1	-0.3 (-2.9, 2.3)	**0.42**	-	-	
**Subgroup analyses statin intervention on BMD**						
**Overall effect**	14	-0.06 (-0.08, -0.04)	**<0.001**	<0.001	97.7%	
**Trial location**						

Denmark	6	-0.002 (-0.01, 0.008)	**0.6**	0.2	20.2%	<0.001
USA	2	-0.4 (-0.5, -0.2)	**<0.001**	<0.001	93.8%	
China	2	0.009 (-0.007, 0.02)	**0.2**	<0.001	96.1%	
Thailand	1	0.05 (0.04, 0.07)	**<0.001**	-	-	
**Study design**						
Parallel	14	-0.06 (-0.08, -0.04)	**<0.001**	<0.001	97.7%	<0.001
Crossover	0	-	**-**	-	-	
**Solubility**						
Lipophilic	14	-0.06 (-0.08, -0.04)	**<0.001**	<0.001	97.7%	-
Hydrophilic	0	-	**-**	-	-	
**Sex**						
Both sexes	1	0.05 (0.04, 0.07)	**<0.001**	<0.001	-	<0.001
Female	10	-0.1 (-0.2, -0.08)	**<0.001**	0.001	97.9%	
Male	3	0.009 (-0.007, 0.02)	**0.2**	0.88	96.1%	
**Trial duration (weeks)**						
≤50	3	0.009 (-0.007, 0.02)	**0.2**	<0.001	96.1%	<0.001
>50	11	-0.1 (-0.1, -0.06)	**<0.001**	0.03	98%	
**Baseline age (yrs)**						
≤60	5	-0.3 (-0.5, -0.06)	**0.78**	0.01	98.8%	0.01
>60	9	-0.01 (-0.003, 0.02)	**0.03**	0.1	92.3%	
**Statin types**						
Simvastatin	7	-0.002 (-0.01, 0.008)	**0.6**	0.2	20.2%	<0.001
Atorvastatin	7	-0.1 (-0.2, -0.1)	**<0.001**	<0.001	98.8%	
**BMD sites**						
Lumbar spine	6	-0.2 (-0.3, -0.1)	**<0.001**	<0.001	98.9%	<0.001
Total hip	1	0.01 (-0.01, 0.04)	**0.2**	0.01	84.2	
Femoral neck	3	-0.002 (-0.007, 0.003)	**0.4**	0.9	0.0%	
Interthrochanter	1	0.02 (-0.008, 0.05)	**0.1**	-	-	
Throchante	2	0.0 (-0.02, 0.02)	**1**	-	-	
Wards	1	-0.02 (-0.04, -0.002)	**0.03**	-	-	
Forearm	9	0.05 (0.04, 0.07)	**<0.001**	-	-	
**Quality of studies**						
Poor	3	0.009 (-0.007, 0.02)	**0.45**	0.2	96.1%	0.06
Fair	5	-0.3 (-0.6, -0.01)	**0.15**	0.04	99.2%	
Good	6	-0.002 (-0.01, 0.008)	**0.6**	<0.001	20.2%	
**Subgroup analyses statin intervention on CTX**						
**Overall effect**	8	0.01 (-0.03, 0.06)	**0.5**	0.001	71.2%	
**Trial location**						
Denmark	1	-9.4 (-168, 186)	**0.9**	<0.001	-	
USA	2	22.5 (5.6, 39.4)	**0.009**	0.8	0.0%	
Germany	1	-0.006 (-0.1, 0.1)	**0.9**	0.38	-	<0.001
New Zealand	1	0 (-0.06, 0.06)	**1**	-	-	
UK	1	-0.04 (-0.08, -0.0)	**0.04**	-	-	
Thailand	1	0.06 (0.007, 0.1)	**0.02**	-	-	
China	1	0.04 (-0.1, 0.1)	**<0.001**	-	-	
**Study design**						
Parallel	7	-0.02 (-6.5, 0.8)	**0.7**	0.06	58.8%	0.4
Crossover	1	0 (-0.06, 0.06)	**1**	-	-	
**Solubility**						

Lipophilic	8	0.01 (-0.03, 0.06)	**0.5**	0.001	71.2%	-
Hydrophilic	0	-	**-**	-	-	
**Sex**						
Both sexes	3	0.007 (-0.05, 0.07)	**0.8**	<0.001	76.5%	0.2
Female	4	11.2 (-5.5, 27.9)	**0.1**	0.67	56.8%	
Male	1	0.04 (0.02, 0.05)	**<0.001**	-	-	
**Trial duration (weeks)**						
≤12	5	-0.01 (-0.08, 0.04)	**<0.001**	<0.001	0%	0.08
>12	3	0.04 (0.03, 0.05)	**0.5**	0.08	51.3%	
**Baseline age (yrs)**						
≤60	4	-0.02 (-0.1, 0.07)	**0.6**	0.6	0%	0.1
>60	4	0.04 (0.03, 0.05)	**<0.001**	0.04	62.9%	
**Statin types**						
Fluvastatin	1	9.4 (-168.1, 186)	**0.9**	-	-	0.20
Simvastatin	4	0.01 (-0.1, 0.1)	**0.8**	0.002	80.5%	
Atorvastatin	3	0.03 (0.01, 0.05)	**0.02**	0.3	12.1%	
**Quality of studies**						
Poor	4	11.2 (-5.4, 27.9)	**0.1**	0.12	56.6%	0.4
Fair	3	-0.006 (-0.1, 0.1)	**0.8**	0.002	76.5%	
Good	1	0.007 (-0.05, 0.07)	**0.9**	-	-	
**Subgroup analyses statin intervention on NTX**						
**Overall effect**	6	-0.2 (-0.5, 0.1)	**0.1**	0.3	12.1%	
**Trial location**						
USA	5	-0.1 (-0.5, 0.2)	**0.5**	0.2	25%	0.5
UK	1	0.3 (-0.8, 0.1)	**0.1**	-	-	
**Study design**						
Parallel	6	-0.2 (-0.5, 0.1)	**0.1**	0.3	12.1%	-
Crossover	0	-	**-**	-	-	
**Sex**						
Both sexes	4	-0.3 (-0.6, 0.01)	**0.06**	0.8	0%	0.41
Female	2	0.2 (-1.08, 1.4)	**0.7**	0.07	69.3%	
**Trial duration (weeks)**						
≤12	6	-0.2 (-0.5, 0.1)	**0.1**	0.3	12.1%	-
>12	0	-	**-**	-	-	
**Baseline age (yrs)**						
≤60	6	-0.2 (-0.5, 0.1)	**0.1**	0.3	12.1%	
>60	0	-	**-**	-	-	
**Statin types**						
Simvastatin	5	-0.1 (-0.5, 0.2)	**0.3**	0.2	29.4%	-
Pravastatin	1	-0.2 (-1.05, 0.4)	**0.4**	-	-	
**Solubility**						
Lipophilic	5	-0.1 (-0.5, 0.2)	**0.3**	0.2	29.4%	-
Hydrophilic	1	-0.2 (-1.05, 0.4)	**0.4**	-	-	
**Quality of studies**						
Poor	2	0.2 (-1.08, 1.4)	**0.7**	0.07	69.3%	0.4
Fair	4	-0.3 (-0.6, 0.01)	**0.8**	0.8	37.8%	
Good	0	-	**-**	-	-	
**Subgroup analyses statin intervention on osteocalcin**						

**Overall effect**	7	0.1 (-0.2, 0.5)	**0.4**	0.007	66.3%	
**Trial location**						
Denmark	1	-0.06 (-0.5, 0.4)	**0.8**	0	-	
New Zealand	1	-0.2 (-0.8, 0.2)	**0.3**	0	-	
USA	3	0.1 (-0.5, 0.7)	**0.7**	0.1	56.4%	0.01
UK	1	0.08 (-0.4, 0.5)	**0.7**	0	-	
China	1	1.02 (0.4, 1.5)	**<0.001**	0	-	
**Study design**						
Parallel	6	0.1 (-0.8, 0.2)	**0.6**	0.2	34.6%	-
Crossover	1	-0.2 (-0.8, 0.2)	**0.3**	-		
**Statin types**						
Fluvastatin	1	-0.6 (-0.5, 0.4)	**0.8**	-		
Atorvastatin	2	0.3 (-0.9, 1.6)	**0.5**	0.001	90.9%	
Simvastatin	3	0.09 (-1.03, 1.2)	**0.8**	0.03	78.1%	0.03
Pravastatin	1	0.1 (-0.6, 0.9)	**0.7**	-		
**Solubility**						
Lipophilic	6	0.1 (-0.2, 0.6)	**0.4**	0.003	71.9%	
Hydrophilic	1	0.1 (-0.6, 0.9)	**0.7**	-	-	
**Trial duration (weeks)**						
≤12	5	0.4 (-0.5, 1.5)	**0.3**	0.005	87.6%	0.4
>12	2	0.004 (-0.3, 0.3)	**0.9**	0.2	33%	
**Sex**						
Both sexes	5	0.004 (-0.3, 0.3)	**0.9**	0.2	33%	
Female	1	-0.06 (-0.5, 0.4)	**0.8**	-	-	0.004
Male	1	1.02 (0.4, 1.5)	**<0.001**	-	-	
**Baseline age (yrs)**						
≤60	5	0.004 (-0.3, 0.3)	**0.9**	0.005	87.6%	0.4
>60	2	0.4 (-0.5, 1.5	**0.3**	0.2	33%	
**Quality of studies**						
Poor	1	-0.06 (-0.5, 0.4)	**0.8**	-	-	
Fair	5	0.2 (-0.3, 0.8)	**0.4**	0.002	75.8%	0.7
Good	1	0.08 (-0.4, 0.5)	**0.7**	-	-	

Abbreviations: **T2DM**: Type 2 diabetes mellitus, **NAFLD**: Non-alcoholic fatty liver disease, **MetS**: Metabolic syndrome, **HTN**: Hypertension, **NAFLD**: Non-alcoholic fatty liver disease, BMI: Body mass index, **yrs**: years, **USA**: United State America.

Table S4. GRADE evidence table for Statin intervention on metabolism markers and mineral density.

Certainty assessment							№ of patients		Effect		Certainty	Importance
**№ of studies**	**Study design**	**Risk of bias**	**Inconsistency**	**Indirectness**	**Imprecision**	**Other considerations**	**[intervention]**	**[comparison]**	**Relative (95% CI)**	**Absolute (95% CI)**		
**ALP**												
8	randomised trials	not serious	not serious	not serious	serious[a]	none	194	197	-	MD **1.1 U/L lower** (2.2 lower to 0.07 lower)	⊕⊕⊕◯ Moderate	IMPORTANT
**BMD**												
14	randomised trials	not serious	very serious[b]	not serious	not serious	dose response gradient	1025	1041	-	MD **0.06 gr/cm 2 lower** (0.08 lower to 0.04 lower)	⊕⊕⊕◯ Moderate	IMPORTANT
**CTX**												
8	randomised trials	not serious	serious[c]	not serious	serious[a]	none	293	249	-	MD **0.01 higher** (0.03 lower to 0.06 higher)	⊕⊕◯◯ Low	IMPORTANT
**NTX**												
6	randomised trials	not serious	not serious	not serious	serious[a]	none	88	89	-	SMD **0.2 SD lower** (0.5 lower to 0.1 higher)	⊕⊕⊕◯ Moderate	IMPORTANT
**Osteocalcin**												
7	randomised trials	not serious	not serious[d]	not serious	serious[a]	none	170	149	-	SMD **0.1 SD higher** (0.2 higher to 0.5 higher)	⊕⊕⊕◯ Moderate	IMPORTANT

Explanations: a. The optimal information size was not reached, downgraded, b. Serious inconsistency since I2=97.7%. Downgrade, c. Serious inconsistency since I2=71.2%. Downgrade, d. Serious inconsistency since I2=66.3%. However, the value of I was < 50% in the subgroup of trials with double-blinded, and the significance, direction, and magnitude of the effect remained unchanged (MD: 0.1(-0.3, 0.5), N=4, I2=34.6%), not downgrade.

Abbreviations: CI: confidence interval; **MD:** mean difference

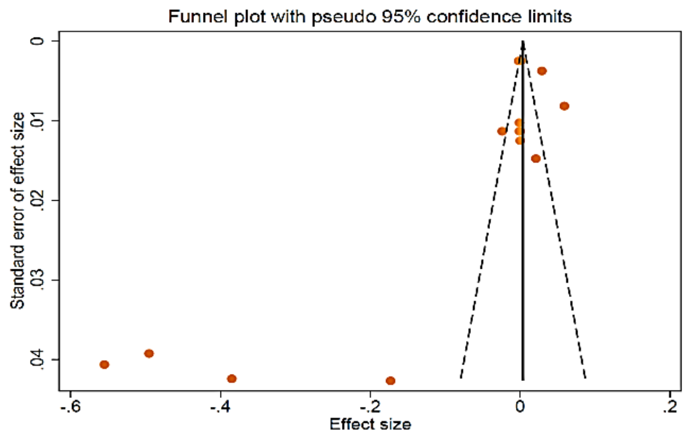

(a)

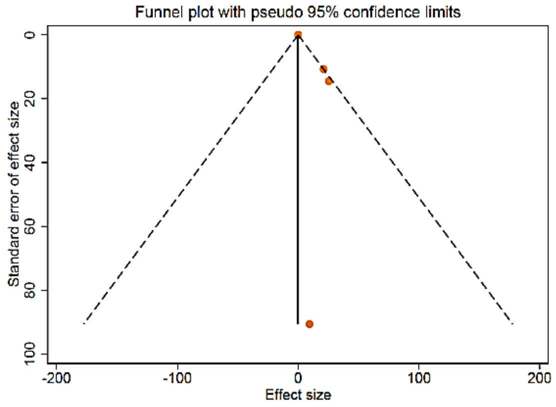

(b)

Figure S1. Funnel plots (with pseudo 95% CIs) of the difference in effect size versus the standard error of the effect size for studies that evaluated the effect of statin intervention on BMD (a), and CTX (b).

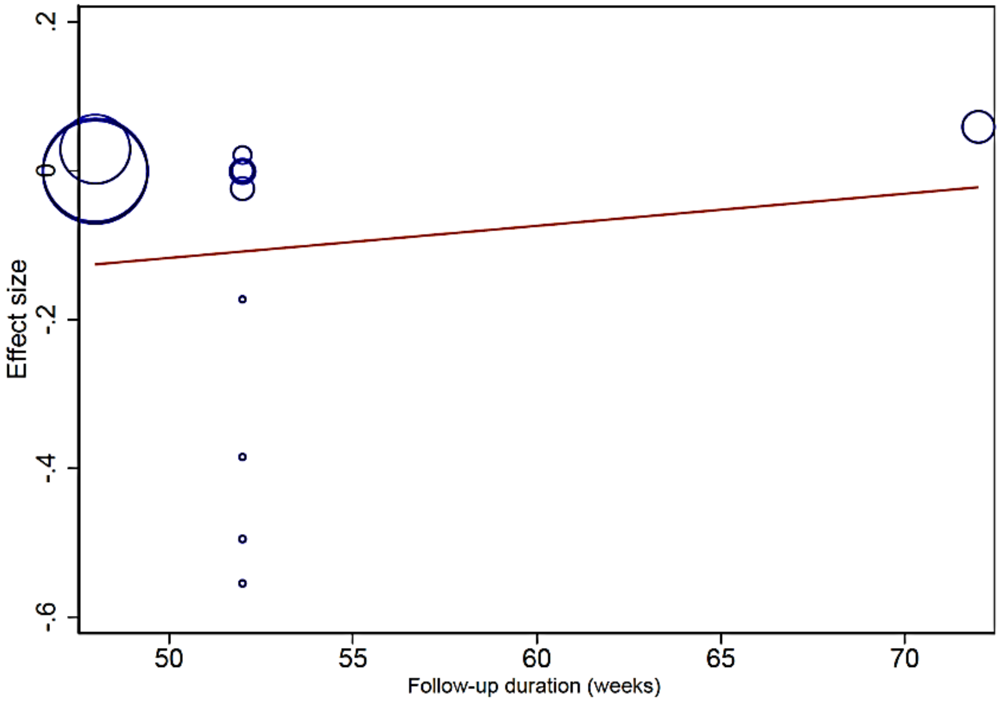

Figure S2. Random-effects meta-regression plots of the association between WMD of BMD following Statin intervention (Duration).

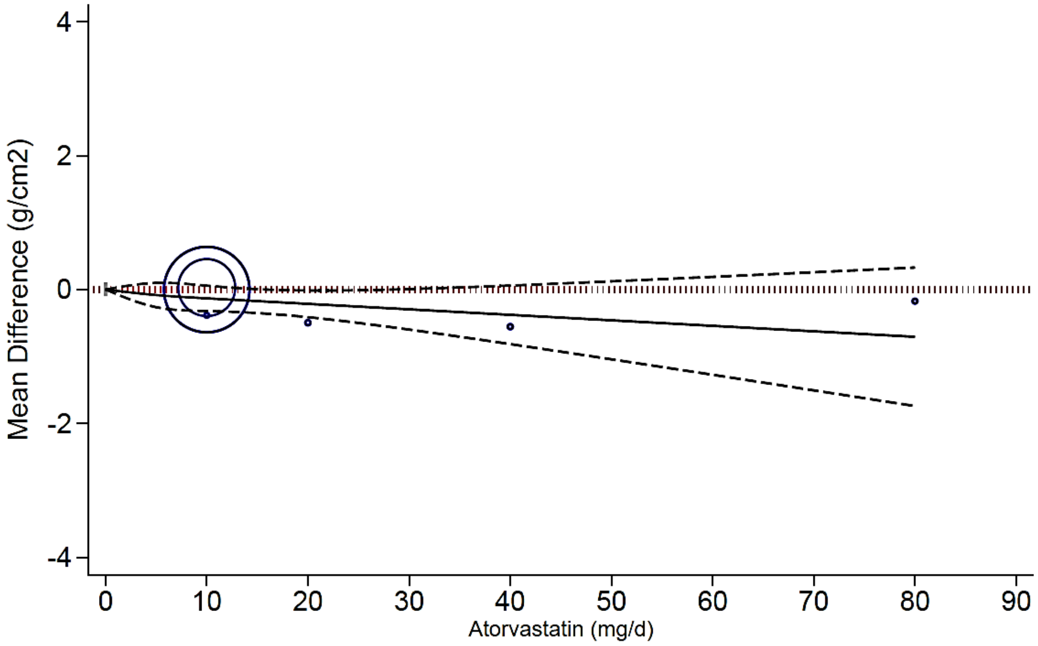

$P_{dose\text{-}response}$ = 0.108; $P_{non\text{-}linearity}$ = 0.729

Figure S3. Dose-dependent effect of Statin intervention on BMD levels.
